# Transcatheter mitral valve-in-valve for pregnancy with anti-phospholipid syndrome: a case report

**DOI:** 10.1186/s13019-024-02702-1

**Published:** 2024-06-19

**Authors:** Zhenzhong Wang, Yuxin Li, Shuo Xiao, Qiuji Wang, Zhaolong Zhang, Fengzhen Han, Huanlei Huang

**Affiliations:** 1 Department of Cardiovascular surgery, Guangdong Provincial People’s Hospital (Guangdong Academy of Medical Sciences), Southern Medical University, Guangzhou, 510080 P.R. China; 2 Department of Obstetrics, Guangdong Provincial People’s Hospital (Guangdong Academy of Medical Sciences), Southern Medical University, Guangzhou, P.R. China

**Keywords:** Anti-phospholipid syndrome, Pregnancy, Transcatheter mitral valve-in-valve

## Abstract

**Background:**

Perioperative management and cardiac surgery in pregnant women with anti-phospholipid syndrome combined with heart valve disease have been rarely reported.

**Case presentation:**

We describe a case of transcatheter mitral valve-in-valve replacement in a pregnant woman with bioprosthetic valve failure and anti-phospholipid syndrome at 18 weeks’ gestation. The patient underwent a cesarean section delivery at 34 weeks of gestation, resulting in the birth of a healthy baby.

**Conclusions:**

Transapical mitral valve-in-valve surgery resulted in safe maternal and infant outcomes in a pregnant woman with anti-phospholipid syndrome combined with mitral bioprosthetic valve failure. The success of this procedure underscored the importance of multidisciplinary teamwork.

**Supplementary Information:**

The online version contains supplementary material available at 10.1186/s13019-024-02702-1.

## Background

Cardiac surgery during pregnancy has always been challenging due to the inherent difficulties and risks involved. There are very few cases wherein surgeons successfully performed interventional procedures during pregnancy [1, 2]. Particularly, this challenge can be seen in patients who also have anti-phospholipid syndrome (APS), a systemic autoimmune disorder with vascular and obstetric manifestations, including venous thromboembolism, stroke, recurrent early miscarriages, and late pregnancy losses [3]. Fetal mortality could reach up to 27.1% in patients with known APS [4]; even if APS is treated with heparin and low-dose aspirin, fetal mortality could reach up to 10–12% [5]. Performing repeat cardiac surgery in such a complex situation to achieve safe maternal and infant outcomes is extremely rare. Presenting this case, we opt for offering insights into medical care experiences in patients facing similar situations during the perinatal period.

## Case presentation

A 35-year-old woman, gravida two para zero, at 18^+ 4^ weeks of gestation presented with shortness of breath and palpitations. In 2018, she underwent double bioprosthetic valve replacement (21 mm Carpentier-Edwards Perimount aortic prosthesis and 25 mm Carpentier-Edwards Perimount prosthesis), tricuspid valvuloplasty (30 mm Carpentier-Edwards MC3), and atrial fibrillation (AF) ablation due to rheumatic cardiac valve disease. Concurrently, she was diagnosed with APS and administered hydroxychloroquine (200 mg/day for) and methylprednisolone (4 mg/day for). Due to APS, the patient experienced difficulty conceiving naturally, with two failed artificial inseminations in 2019 and 2020. Of note, her first test-tube baby ended in an abortion in June 2021 at 8 weeks of gestation due to an unexplained silent miscarriage. Low-molecular-weight heparin (LMWH; 0.6 mg twice daily) was initiated from the sixth week of gestation.

**Fig. 1:**
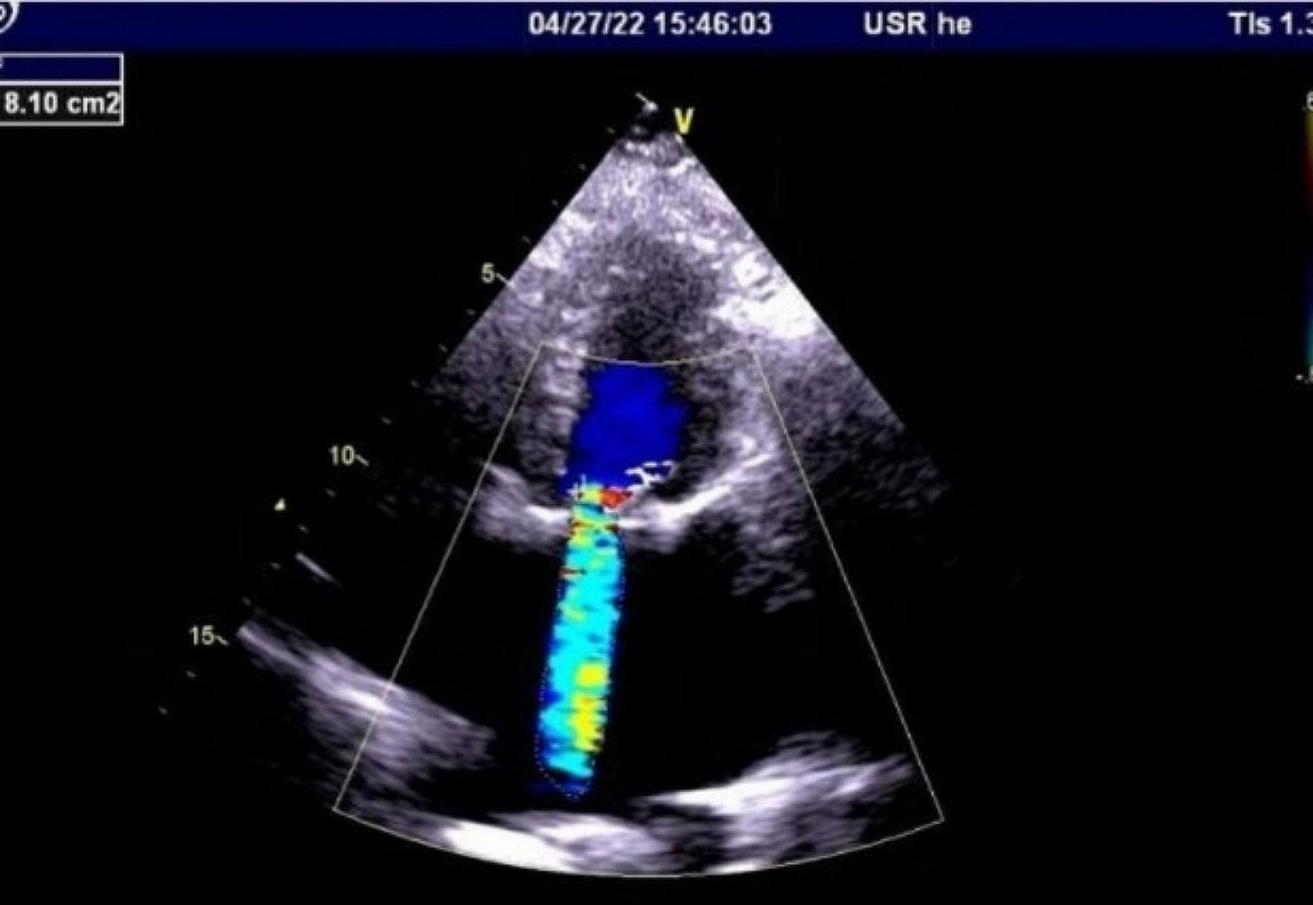
Preoperative transthoracic echocardiogram: The top left corner shows the measured reflux area of 8.1cm2

A coagulation test showed a slight increase in fibrinogen and D-dimer levels without other abnormalities. Blood tests for rheumatic immune indicators revealed anti-nuclear antibody (+); anti-cardiolipin antibody, IgA 53.7 APL/ml; anti-β2-glycoprotein I antibody, IgA 56.3 U/ml; and anti-SSA/Ro60 antibody (+++). Preoperative echocardiography showed severe intravalvular regurgitation (color flow jet area, 8.1 cm^2^; mean gradient, 17.3 mmHg) and severe pulmonary artery hypertension (PAH; 91 mmHg) (Fig. 1). Fetal ultrasonography confirmed a healthy fetus.

Transcatheter mitral valve-in-valve (TMViV) was performed in a hybrid operating room. Anesthesia was administered according to the principles of general anesthesia during pregnancy. To minimize radiation exposure to the fetus, the patient’s abdomen was wrapped 360° with a lead coat, and fetal electrocardiogram was performed by an obstetrician.

**Fig. 2:**
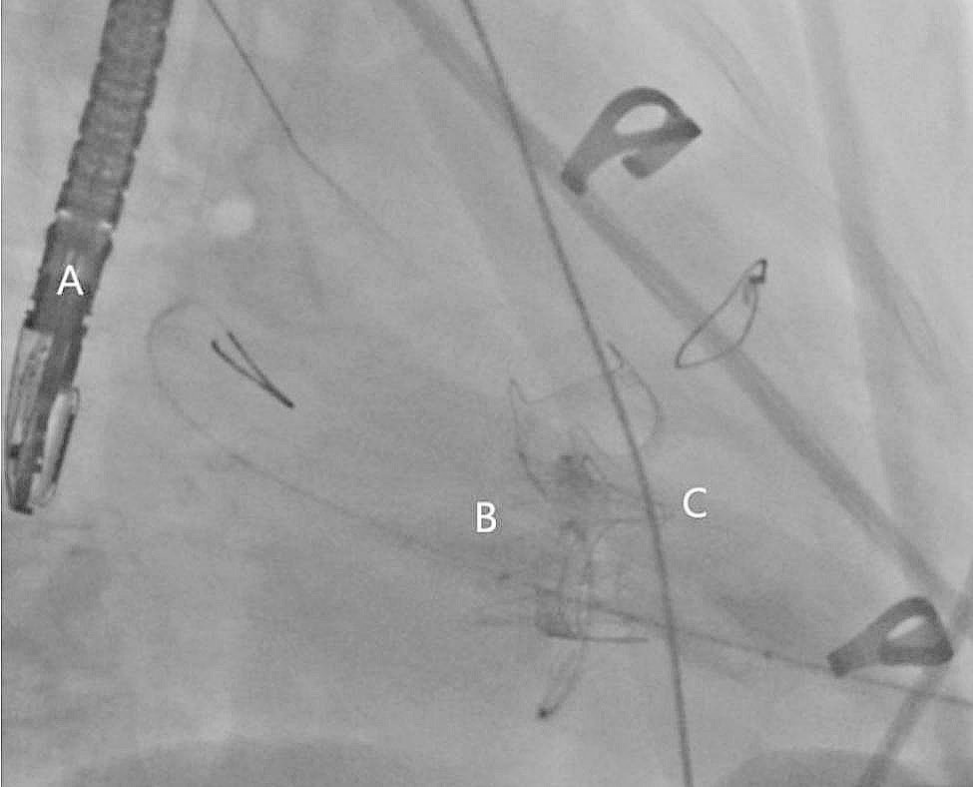
Fluoroscopic image depicting (**A**) transesophageal echocardiogram probe, (**B**) 25 mm J-valve, and (**C**) mitral bioprosthetic valve failure

**Fig. 3:**
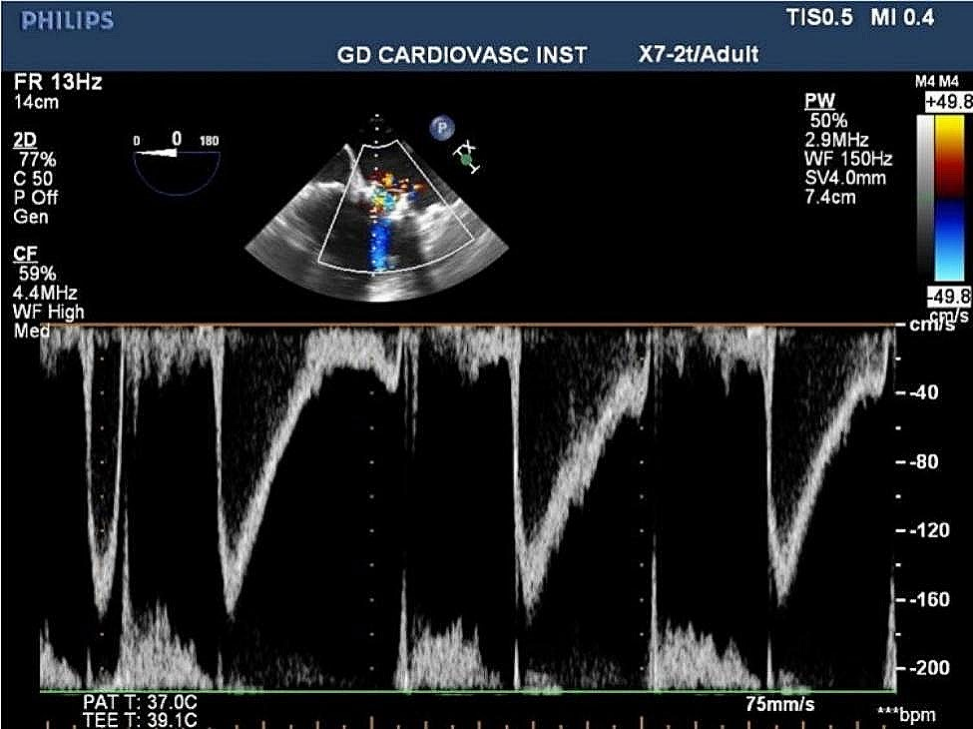
Postoperative transesophageal echocardiogram showed no significant mitral regurgitation

A temporary endocardial pacing lead was inserted into the right internal jugular vein. The left ventricular apex was located under digital subtraction angiography (DSA) and transesophageal echocardiography (TOE) guidance. A 4-cm incision was made in the fifth intercostal space. Heparin was administered to achieve an activated clotting time (ACT) of ≥ 250; the actual ACT reached 450. After exposing the apex, double layers of a 2 − 0 prolene apical purse with a spacer were punctured at the midpoint. Guidewires were placed through the mitral valve into the left atrium, a 6Fr vascular sheath was inserted. Thereafter, the super-hard guidewire was exchanged carefully to avoid wrapping the mitral chordae tendineae. A support track across the original mitral valve bioprosthesis was formed, for which a 20Fr vascular sheath was used to dilate it. During paused breathing and a paced heart rate of 140 beats/min, a 25 mm J-valve (JieCheng Medical Technology Corporation Ltd., Suzhou, China) was delivered (Fig. 2). Breathing was resumed after the valve was released. The fetal heart rate did not decrease significantly. The valve position was determined using DSA and TOE; the mitral valve was not fully expanded. The shape of the valve after dilation with a 25Fr balloon was more appropriate. The J-valve system was released; the purse string was tightened; and protamine was administered for heparin neutralization. A drainage tube was placed to avoid tamponade. Postoperative TOE revealed a mean mitral valve gradient of 2 mmHg without obvious valvular regurgitation (Fig. 3). The systolic blood pressure remained > 70 mmHg throughout the procedure. Ichthyosin (50 mg) was used to neutralize the heparin. The overall operating time was 127 min. Moreover, no contrast agent was administered during the procedure. The endotracheal tube was removed in the operating room.

The patient was discharged three days after surgery. Fetal and transthoracic ultrasounds were performed at least every two weeks, and rheumatic immune indicators were tested monthly. No significant changes were observed in postoperative blood test results for rheumatic immunity. With the approval of the rheumatologist, hydroxychloroquine (200 mg), methylprednisolone (4 mg), aspirin (100 mg) and LMWH (0.6 ml: 6000iu) were administered daily. At 29 weeks of gestation, aspirin was discontinued, and LMWH dosage was changed into 0.4 ml. At 33^+ 3^ weeks of gestation, the patient was diagnosed with threatened premature labor. Echocardiography revealed a left ventricular ejection fraction of 40% and a PAH of 50 mmHg. After a multidisciplinary team consultation, a cesarean section was performed due to the high maternal and fetal risks of continued pregnancy. The patient gave birth to a healthy baby at 34^+ 1^ weeks, and the APGAR scores of the newborn were 10 at both the 1st and 5th min. Echocardiographic and electrocardiographic data before surgery, after surgery, before delivery, after delivery, and at the most recent time points are presented in Table 1.

**Table 1:** Comparison of echocardiographic and electrocardiographic data

	Pre-operation	Post-operation	Pre-delivery	Post-delivery	Latest
Color flow jet area of mitral valve (cm^2^)	8.1	No obvious regurgitation was observed	No obvious regurgitation was observed	3.9	1.8
Color flow jet area of tricuspid valve (cm^2^)	3.3	3.0	4.4	4.5	1.9
Peak velocity of aortic valve (m/s)	3.2	3.1	2.6	2.6	3.4
Left atrium (mm)	69	51	48	64	63
Left ventricular ejection fraction (%)	63	70	43	50	53
Pulmonary artery systolic pressure (mmHg)	91	38	41	44	29
Electrocardiogram	AF, 152 bpm	AF, 76 bpm	AF, 127 bpm	AF, 70 bpm	AF, 87 bpm

*Abbreviation* AF, atrial fibrillation; bpm, beats per minute

## Discussion and conclusions

A 38-year-old pregnant woman at 15 weeks of gestation with an unknown primary APS underwent tricuspid valve tumor resection under cardiopulmonary bypass (CPB). A case, as reported in the literature, underwent a cesarean section at 35 weeks’ gestation, giving birth to a healthy fetus [6, 7]. The biggest difference between the two cases is that our case involved recurrent cardiac surgery. Approximately 20% of fetal losses are caused by CPB [8]. In addition, reoperation is a risk factor for maternal and fetal mortality [9]. The most obvious advantage of TMViV over other methods is that it does not require CPB. Moreover, obstetric morbidity in patients with APS includes early recurrent miscarriage, unexplained fetal loss, and/or premature birth [10]. The patient did not give consent to undergo abortion before cardiac surgery. The main reason for choosing a surgical method was to maximize the duration of the gestation period, thereby increasing the probability of fetal survival. Considering the potential for reoperation and the value of the “precious child”, TMViV was considered the most viable approach to ensure the safety of both the patient and fetus, mainly due to its avoidance of CPB and shorter operative time. However, this could carry risks and pressures. Surgeons must be highly experienced, given the lack of preoperative examination using computed tomography and magnetic resonance imaging. If a miscarriage occurs after surgery, the patient would undergo a third cardiac surgery due to bioprosthetic valve failure, diminishing the significance of the initial cardiac surgery.

The surgical technique itself was not the most difficult process, minimizing additional risks was a key factor. First, a semi-monthly multidisciplinary team consultation was conducted regarding patients’ APS antibody levels and cardiac ultrasound findings. The patients had a direct access to a multidisciplinary team of physicians at any time via telephone or social media. Cardiac surgery was the most recommended procedure time to perform. Second, abdominal radiological isolation was necessary to reduce the risk of fetal radiation exposure. Third, we ensured that the mother’s systolic blood pressure was > 70 mmHg, including that during valve release, and recommended elevating the blood pressure with phenylephrine. Fourth, if the fetus showed signs such as a slow heart rate, atosiban could have been considered [11]. Fifth, it was important to adjust the anticoagulant regimen according to the rheumatologist. To avoid thrombosis and reduce the risk of adverse fetal outcomes, we used aspirin and LMWH therapy during pregnancy, as recommended [3]. Finally, the patient was administered a small oral dose of diuretics postoperatively to avoid amniotic fluid reduction due to hypovolemia.

TMViV might be an effective method to ensure the safety of mothers and fetuses in pregnant cardiac patients with APS who require heart valve reoperation and strongly deny abortion.

### Electronic supplementary material

Below is the link to the electronic supplementary material.

Supplementary Material 1

## Data Availability

All data generated or analysed during this study are included in this published article.

## Abbreviations

ACT: activated clotting time
AF: atrial fibrillation
APS: anti-phospholipid syndrome
CPB: cardiopulmonary bypass
DSA: digital subtraction angiography
LMWH: low-molecular-weight heparin
PAH: pulmonary artery hypertension
TMViV: transcatheter mitral valve-in-valve
TOE: transesophageal echocardiography
